# Influences of TiO_2_ and SiO_2_ nanofillers on the hardness and surface roughness of maxillofacial silicone elastomers after two years of outdoor tropical weathering: An in vitro study

**DOI:** 10.1371/journal.pone.0344522

**Published:** 2026-04-15

**Authors:** Mohammed Mousa, Belal Elmarhoumy, Mahmoud Salloum, Abdalwhab Zwiri, Mohammed Sghaireen, Thamir Bahattab, Ahmed Almutairi, Njoud Almuzaini, Johari Abdullah

**Affiliations:** 1 Department of Prosthetic Dental Sciences, College of Dentistry, Jouf University, Sakakah, Jouf, Saudi Arabia; 2 Prosthodontic Unit, School of Dental Sciences, Universiti Sains Malaysia, Kubang Kerian, Kota Bharu, Kelantan, Malaysia; 3 Department of Prosthodontics, Faculty of Dentistry, Sinai University, Kantara Branch, Ismailia, Egypt; 4 Department of Oral Surgery and Diagnostic Sciences, Faculty of Dentistry, Applied Science Private University, Amman, Jordan; 5 Dental Research Unit, Center for Transdisciplinary Research (CFTR), Saveetha Dental College, Saveetha Institute of Medical and Technical Sciences, Saveetha University, Chennai, India; University of Sharjah, UNITED ARAB EMIRATES

## Abstract

The properties of maxillofacial silicone elastomers (MFSE) are far from ideal and still require reinforcement. The incorporation of nanofillers (NFs) at high levels still requires further investigation. This study evaluated the influence of different percentages (3 and 10% w/w) of titanium dioxide (TiO_2_) and silica (SiO_2_) NFs on the roughness and hardness of A-2000 silicone elastomer after two years of natural weathering. A total of 80 accepted specimens were divided into two groups: weathered and non-weathered. Each group was equally subdivided into 5 subgroups: non-filled, filled with 3% SiO2, 10% SiO2, 3% TiO_2_, and 10% TiO_2_. Surface roughness was quantitatively measured using a profilometer and qualitatively using a Scanning Electron Microscope (SEM). The hardness was measured using a digital Shore-A durometer. The chemical interaction between NFs and elastomers was examined using Fourier Transform Infrared (FTIR) spectroscopy. The data were exported to SPSS, and the analysis was done using 1-way ANOVA and an independent t-test. Adding 10% SiO_2_ and TiO_2_ NFs significantly decreased the surface roughness of A-2000 MFSE, with no significance among all concentrations after 2-year natural weathering (*P* = 1). 10% SiO_2_ was associated with the highest hardness of A-2000 MFSE (32.7 ± .34; *P* < .001). The hot, humid natural weather conditions demonstrate a significant decrease in surface roughness and an increase in the hardness of A-2000 MFSE (*P* < .001). FTIR analysis confirmed that there were no changes in the chemical interactions between the silicone matrix with and without NFs.

## Introduction

Maxillofacial silicone elastomers (MFSEs) are used to treat patients with developmental and congenital abnormalities or impairments brought on by surgery or trauma [1]. For patients requiring maxillofacial prostheses, the aim is to achieve surfaces with low friction and minimal skin deformation, facilitating smooth gliding and enhancing patient comfort while reducing irritation. On the other hand, other scenarios require optimized grip performance, controlled by static friction behaviour and distinct skin deformation characteristics [2]. Facial skin exhibits an arithmetic mean roughness (R_a_) typically spanning 6–14 μm. Clinical studies confirm that anatomical site, age, and gender are key variables influencing variations in skin roughness across facial regions [3,4]. The ideal materials should be stable with premium physical and mechanical properties [5]. Among the various external maxillofacial prosthetic materials, including wood, ivory, wax, and metals, despite their widespread use, MFSEs have limitations that hinder long-term clinical success [6]. Based on methods of polymerization, the MFSEs are divided into high- and room-temperature vulcanizing silicones. As one of the room-temprature vulcanizing silicone, A2000 is among the most used MFSEs [5]. However, delamination of the retentive substrate, degradation of mechanical and physical characteristics, and color instability were cited as reasons for this shortcoming [7–9]. Among the reasons for the decline in the mechanical and physical properties of MFSEs, musculature, secretions, and environmental conditions have been well documented in the literature [10–13]. That challenged the researchers to find a way to improve those materials by adding various strengthening fillers to enhance their performance and service life [14].

Interestingly, MFSEs have shown an improvement in their functional, mechanical, and physical properties by adding different percentages of nanofillers (NFs), such as polyester [15], tulles [16], polyamides (Nylon 6) [17], silver-zinc zeolite [18], zirconium silicate (ZrSiO_4_), titanium dioxide (TiO_2_), Zirconia dioxide (ZrO_2_), Silica dioxide (SiO_2_), Titanium Silicate (TiSiO_4_), zinc oxide (ZnO), and cerium oxides (CeO_2_) [14,19]. These NFs were found to optimize the biological, optical, and mechanical characteristics of MFSEs due to their diverse properties, such as small size, high surface area, and active interactions with the matrix [20]. However, no universal filler can be used with different silicone elastomers. In the last 10 years, tremendously valuable studies have evaluated the influence of NFs on the performance of MFSEs after polymerization and aging [19,21–32].

Apart from the mechanical properties, surface roughness and hardness are among the critical surface properties of the MFSEs. Surface roughness is the surface irregularities measured in 2-dimension (using a Stylus Profilometer) or 3-dimension (using an Optical Profilometer or Atomic Force Microscopy) [13]. Surface roughness is considered an essential feature for achieving prosthesis retention or mimicking the skin texture and wrinkles of the patient. However, the rough surface provokes bacterial adherence as it demonstrates difficulty in cleaning [13]. The hardness of silicone as a surface characteristic is its resistance to indentation, scratching, cutting, and mechanical wear, ensuring the durability of the MFSE. The importance of hardness in MFSEs comes from the resistance of a material to permanent surface deformation. That increases the service quality and increases the durability of the prosthesis [33].

One of the essential factors affecting the performance of MFSEs is the chemical interaction of the fillers and the matrix. No chemical reaction between the fillers and the silicone matrix should have occurred, as such a reaction would compromise the polymerization reaction and devastate the physical and mechanical properties of the materials [34]. Fourier Transform Infrared (FTIR) is a non-destructive technique used effectively for the analysis of biological specimens by measuring the intensity of light absorbed or transmitted by a sample at specific wavelengths [35]. By analyzing this absorption, information about the concentration, composition, or structure of the substance can be obtained [36]. FTIR is a rapidly expanding research area that uses infrared spectroscopy of solids, liquids, or gases, with a focus on cytological and histological diagnosis through spectral images. It can also be used to monitor the ageing of silicone elastomers. Each material has a range and its own FTIR fingerprint [27,37,38].

Although the influence of adding different fillers has been extensively covered in the literature, to the knowledge of the authors, no study has examined the impact of high filler content on the surface properties of one of the most important MFSEs, A-2000. Besides, A-2000 is less studied under long-term natural weathering, which provides long-term follow-up on the clinical use of prostheses fabricated from these materials. This study aimed to evaluate the influences of adding different percentages of SiO_2_ and TiO_2_ (3 and 10% w/w) on the surface roughness and hardness of the A-2000 MFSE after two years of natural weathering. The null hypotheses for this study were that there are no significant differences in adding SiO_2_ and TiO_2_ NFs to the surface roughness and surface hardness of A-2000 MFSE, and no chemical reaction between NFs and the MFSEs within the sensitivity limits of FTIR analysis.

## Materials and methods

### Design and sampling

The current study was an in vitro study conducted in the School of Dental Sciences, Universiti Sains Malaysia. Using G* Power software, 80 specimens of room-temperature-vulcanised A-2000 MFSE (Factor II, Inc., Lakeside, Arizona, USA) were prepared. The sample size was determined using the F test and repeated-measures ANOVA with within-between interactions. Introducing 10 groups, 2 measures (as both roughness and hardness examination were done on the same specimen), β error probability of  .85, α errors of  .05, and effect size of 0.25 [39]. The specimens were prepared according to ISO ASTM D2240 to test the hardness and surface roughness. The specimens were divided into 2 groups, including weathered and non-weathered, with 40 samples for each group. Each group was subdivided in 5 subgroups, with 8 specimens for each subgroups, including MFSEs with no NFs, 3% and 10% SiO_2_ [99 + % SiO_2_ (NPs) with average size 400 nm, Spherical in shape and white with light pink color, and single crystal (MFCD00011232; American Elements, LA, USA)], and 3% and 10% TiO_2_ [99 + % TiO_2_ NFs with average size 800 nm, spherical in shape, and white in color (MFCD00011269; American Elements, LA, USA)].

### Specimen preparations

To prepare the specimens in accordance with ISO ASTM D2240, a stone mold was made using the wax-lost technique. The molds were 10 mm thick and 30 mm in length and width. That was done to prevent sample stacking and to ensure a distance of more than 12 mm from the boundaries to the indenter during the hardness evaluation. Regarding the preparation of the control group, a digital weight scale (Sartorius BSA323S-CW, Göttingen, Germany) was used to weigh 10 grams of base and catalyst (1:1) of A-2000 MFSE materials (Factor II, Incorporated; Lakeside, USA). Both components of the elastomer were manually mixed on silicone mixing pads for 10 minutes, then the mixture was placed in a Hoover (Mixyvac t; Manfredi, Pinerolo, Italy) to provide a homogeneous, air-bubble-free mixture and to reduce agglomeration. The mixture was poured into prepared stone molds using a low-speed vibrator to ensure it contained no air bubbles. The stone molds with the specimens were left in a 660-psi hydraulic press (Silfradent Co., Sofia (FC), Italy) for 72 hours at room temperature (~30 °C) to allow silicone polymerization. The specimens were carefully removed from their molds, and excess material was precisely snipped away. The specimens were examined visually and tactilely, and those with no tears, nicks, or interior flaws (such as large air bubbles) and whose dimensions met the requirements of ISO ASTM D2240 were included in the study. Specimens that were 10% off the ISO specification or had any apparent flaws were excluded from the study.

For the experimental groups, NFs were weighed at 3% and 10% w/w of the total weight of the silicone elastomer base and catalyst (0.6, 2–3%, and 10%, respectively), added to the silicone foundation (base), and mixed for 20 minutes. The pre-weight catalyst was then added to the base and mixed for 30 minutes using the same mechanical mixer under vacuum. The final specimens were then prepared and refined according to the steps outlined earlier.

One to three days after preparing the specimens, the control specimens were stored in a dark, non-humid box for two years, while the experimental groups were exposed to the natural weather by hanging them on a 45° tilted glassy exposure rack facing the sunrise in the garden of the School of Dental Sciences, Universiti Sains Malaysia, Kelantan, Malaysia. Both weathered and non-weathered specimens were preserved for two years, from December 2021 to December 2023. The specimens were left in place without change in position to expose only one surface. That was done because only one side of the prosthesis faces the natural weather, and that side was assessed for hardness and roughness. The specimens were regularly cleaned with moistened napkins to keep them clean and prevent fungal growth on their surfaces.

Weather parameters were recorded daily on the AccuWeather website (https://www.accuweather.com/). The specimens were cleaned for 10 minutes in natural water (Natural Mineral Water; Spritzer, Perak Darul Ridzuan, Malaysia) and were subsequently wiped, dried, and prepared for examination.

### Surface roughness evaluation

The surface roughness was evaluated quantitatively and qualitatively using a profilometer (Surfcom Flex 50A, Seimitsu Co., Ltd., Tokyo, Japan) and a scanning electron microscope (SEM) (AURA100; Seron Technologies Inc., Gyeonggi, Korea), respectively. When using the profilometer, the machine was calibrated, and the evaluation length, cut-off value, and speed were set to 5.00 mm, 0.80 mm, and 0.30 mm/s, respectively. The surface roughness of each specimen was measured in three different areas, one in the middle and one on each side of the sample. Subsequently, the mean value of the three readings for each specimen was recorded.

Qualitatively, the SEM was used to better investigate the surfaces of the specimens and to assess the distribution and dispersion of NFs within the silicone matrix. The Specimens were prepared by coating them with gold using a sputtering coater (SC7620; Quorum Tech, Laughton, East Sussex, UK) to prevent charging by the electron beam and then mounting them in the machine using the sample holder. The accelerating voltage was set to 10 kV, and the electron beam was aligned to achieve ideal resolution. Then, after the working distance (the distance at which the specimens were apparent on the monitor) was set, the knob was adjusted until the image sharpened (×500 magnification) to cover an area of 100 µm. The high-resolution images were captured and saved as JPEGs, and the test samples were qualitatively compared. Multiple photo captures were selected for each sample, and the highest-roughness photos were selected.

### Hardness evaluation

The hardness was calculated utilizing a digital Shore-A durometer. Each specimen was tested on a hard surface and at 5 different points with a distance of at least 5 mm between adjacent points. The mean hardness values were then calculated as the final value.

### Chemical evaluation of specimens using FTIR

The chemical analysis was carried out on the specimens, which were examined for hardness, using Fourier Transform Infrared spectroscopy with a spectrophotometer (Spectrum 100 PerkinElmer^®^, Waltham, Massachusetts, USA), controlled by PerkinElmer Spectrum Express. After turning the machine on, the instrument was left to warm up to stabilize the light source. The sample compartment was wiped with alcohol gauze to ensure it was dust-free. The control software was turned on, and the machine was calibrated by performing background scanning to measure the baseline spectrum. The samples were prepared and placed into the sample compartment, and the lid was closed to prevent light reflection. Then, their IDs and descriptions were entered into the software, and the spectral range was set to 4000–400 cm ⁻ ¹, the resolution to 4 cm ⁻ ¹, and the number of scans to 34 per spectrum. The mid-region of the spectrum was chosen for the analysis and interpretation of the graphs. The data were analyzed quantitatively and qualitatively using the software that controls the machine. The mid-region was divided into four regions: single bond (2500–4000 cm ⁻ ¹), triple bond (2000–2500 cm ⁻ ¹), double bond (1500–2000 cm ⁻ ¹), and fingerprint (400–1500 cm ⁻ ¹) [37,38].

### Data analysis

The data were analyzed using SPSS version 27.0 (IBM, Armonk, NY, USA). Quantitative data were described as mean and standard deviation, and normality was examined using the Shapiro-Wilk test, so the variables were compared using the Tukey test with multiple 1-way measures ANOVA.

## Results

### Climate data

Table 1 demonstrates the climate data during the exposure time from December 2021 to November 2023. There was a slight variation in the average temperature across months during the year. The average maximum temperature ranged from 29.4 °C in January to 34.2 °C in October, while the average of the minimum temperature ranged from 21.6 °C in October to 25.4 °C in May. The climate of Kelantan shows a high average rainfall, peaking in November (656.1 mm) and lowest in February (97.8 mm), while humidity was very high in November at 88% and lowest in April at 82%. Despite sunrise hours averaging 4.4 to 7.2 per day, the ultraviolet index was very high, ranging from 11 to 14.

**Table 1:** Monthly climatic data and UV index scores during outdoor weathering.

Month 2021–2023	Average temperature Highest/Lowest (F)	Rainfall (mm)	Wind speed (Km/H)	Humidity (%)	Sun Hours / Days	UV index scale
Dec	29.6/ 23.8	436.1	7.66	87	4.4	11
Jan	29.4/23.4	170.6	12.68	84	5.46	12
Feb	30.2/23.4	97.8	10.46	84	6.33	12
March	31.7/24.7	120	11.06	83	6.97	12
April	32.9/24.3	120.9	7.44	82	7.2	12
May	33.8/25.4	144	7.08	83	6.63	12
June	32.8/ 24.1	144.3	7.35	83	6.27	14
July	32.7/24.2	140.5	5.75	84	6.13	14
Aug	32.5/24.3	155.3	6.23	84	6.23	14
Sep	32.2/ 24.3	202.1	6.77	84	6.23	12
Oct	34.2/ 21.6	268.7	6.82	86	5.9	12
Nov	30.4/24.6	656.1	6.87	88	4.6	11

**H/L** Maximum temperature/Minimum temperature; **Km/h** Kilometer per hour, **UV index scale**, 0–2 low, 3–7 moderate, 8 + high to extreme.

### Surface roughness

Table 2 shows the test of normality (Shapiro-Wilk) for both surface hardness and roughness. The test indicates that the data distribution is normal, with no significant deviation from normality.

**Table 2:** Shapiro-Wilk test of normality.

	Materials	Shapiro-Wilk test		
Variable		Statistic	df	Sig.
Surface roughness	NonWeathered_(nonfilled)	.861	8	.122
	Nonweathered_(3%)_Si_filled	.822	8	.049
	NonWeathered_(10%)_Si_filled	.959	8	.798
	NonWeathered_(3%)_Ti_filled	.939	8	.599
	NonWeathered_(10%)_Ti_filled	.967	8	.877
	Weatherd_Nonfilled	.952	8	.736
	Weathered_(3%)_Si_filled	.925	8	.474
	Weathered_(10%)_Si_filled	.980	8	.964
	Weathered_(3%)_Ti_filled	.939	8	.605
	Weathered_(10%)_Ti_filled	.928	8	.502

Regarding surface roughness, Table 3 shows that the analysis of variance (ANOVA) indicates significant differences between groups (SS = 5.6, F = 47.2, *P* < .001, and η2 = .86). The effect is practically substantial, with nearly 86% of the variability attributed to group differences. Table 4 shows the comparison of surface roughness (in µm) of the tested MFSE with different NF concentrations, measured with a profilometer, using Tukey's test. Adding a high percentage (10%) of SiO_2_ and TiO_2_ NF significantly decreased surface roughness in the non-weathered samples (*P* ≤ .001). Adding 10% TiO2 NFs resulted in the lowest roughness, followed insignificantly by 10% SiO_2_ (*P =* 1). There were no significant differences between MFSE with 3% of SiO_2_ and TiO_2_ and other NFs percentages (*P* = 0.94). Adding a low percentage (3%) of TiO2 NF had no impact on surface roughness compared with MFSE without NF (*P* = .54).

**Table 3:** Analysis of variance and mean squares in between and within groups in terms of surface roughness.

ANOVA					
Surface_roughness					
	Sum of Squares	df	Mean Square	F	Sig.
Between Groups	51.110	9	5.679	47.196	.000
Within Groups	8.423	70	.120		
Total	59.532	79			

**Table 4:** The surface roughness (μm) results of the study.

Filler	Non-weathered (Mean ± SD) μm	Weathered (Mean ± SD) μm	*P*-value (Tukey-test)
Non-filled	4.35 ± .63^**[a]^[y]^**^	2.66 ± .26^**[a]^[z]^**^	< 0.001*
3% SiO_2_	3.76 ± .2^**[bc]^[y]^**^	2.3 ± .15^**[ab]^[z]^**^	< 0.001*
10% SiO_2_	3.37 ± .26^**[c]^[y]^**^	1.85 ± .33^**[c]^[z]^**^	< 0.001*
3% TiO_2_	3.99 ± .53^**[ab]^[y]^**^	2.45 ± 0.25^**[ab]^[z]^**^	< 0.001*
10% TiO_2_	3.29 ± .35^**[c]^[y]^**^	2.26 ± .19^**[abc]^[z]^**^	< 0.001*
*P*-value	< .001^**$**^	< .001^**$**^	

^**[a,b,c]**^ The comparison among different groups, while ^**[a]**^ is the highest, ^**[b]**^ is the lowest, and the same letters show no differences.

^**[y,z]**^ There are significant differences within the same group, while ^**[y]**^ is the highest and ^**[z]**^ is the lowest. The same letter/s means no significant differences between the variables

* There are significant differences within the same group.

^**$**^ There are significant differences among different groups.

**Fig 1:**
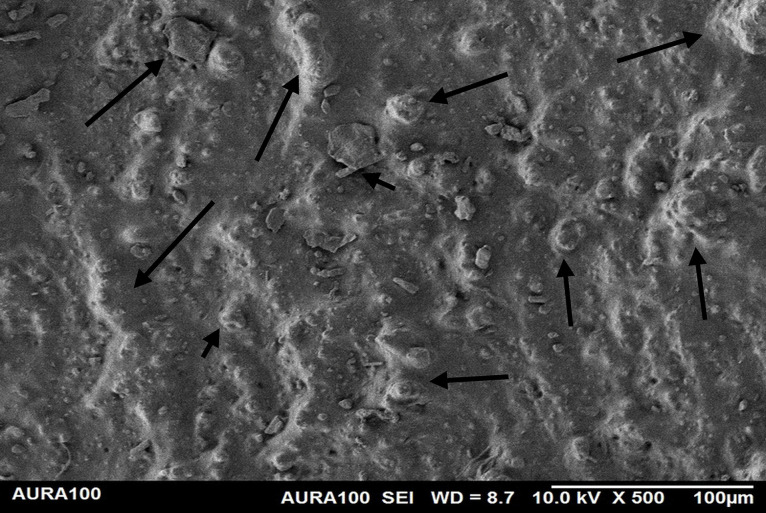
The surface roughness of non-weathered MFSE with no NF.

**Fig 2:**
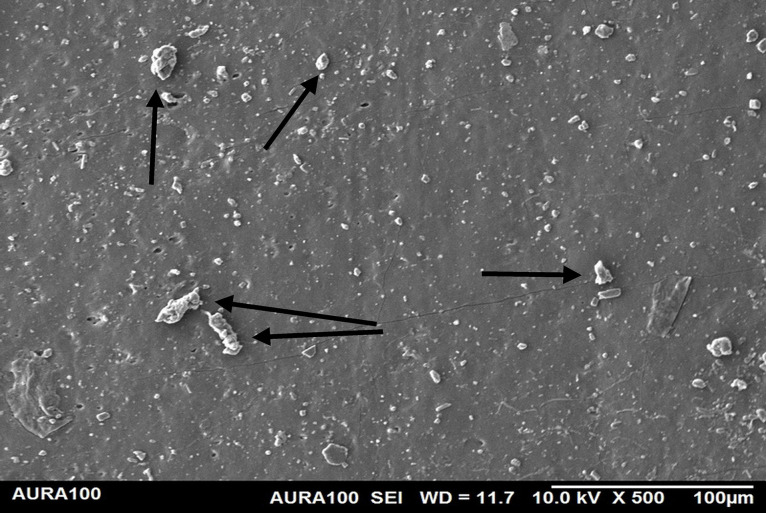
The surface roughness of non-weathered MFSE with 3% SiO_2._

**Fig 3:**
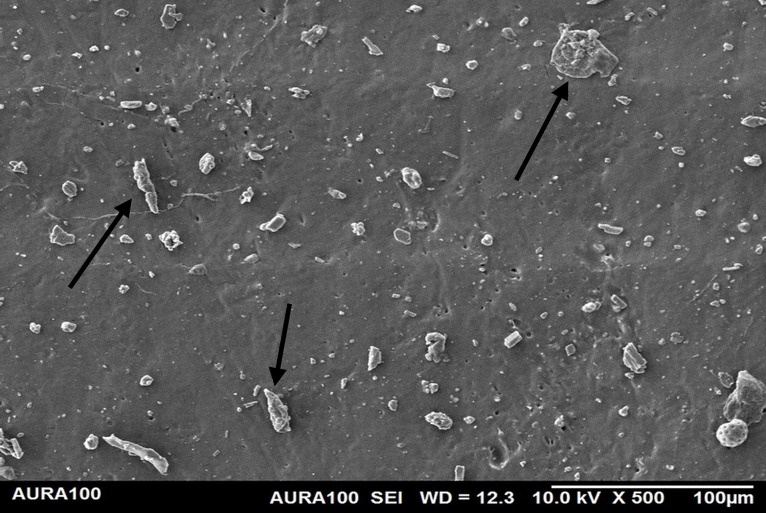
The surface roughness of non-weathered MFSE with 10% SiO_2._

**Fig 4:**
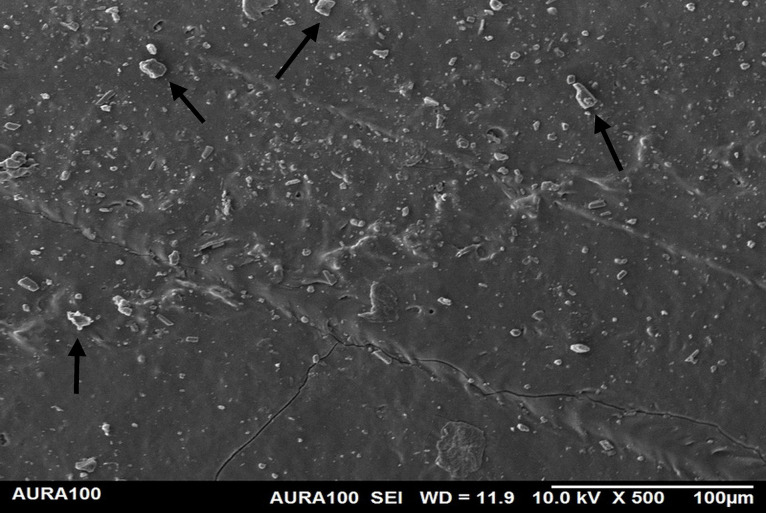
The surface roughness of non-weathered MFSE with 3% TiO_2._

**Fig 5:**
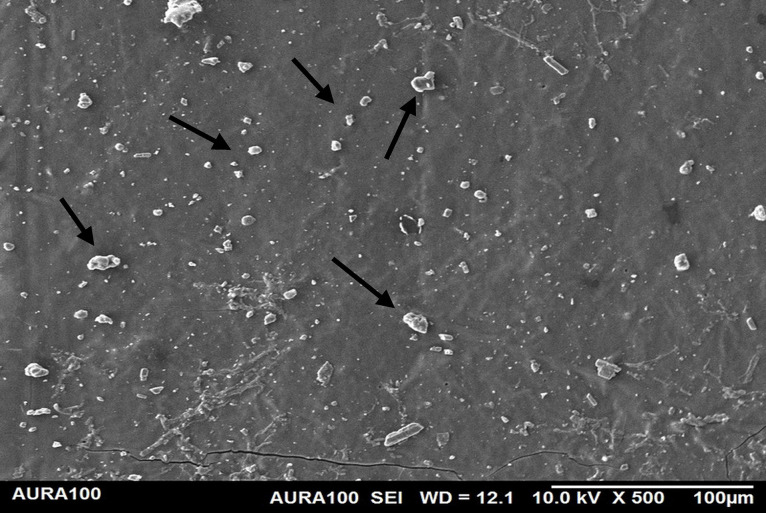
The surface roughness of non-weathered MFSE with 10% TiO_2._

Using the SEM, the non-weathered MFSE with 3% SiO2 showed the least roughness, followed by the 10% TiO2 NF, whereas the non-filled showed the highest roughness. SEM also showed NP agglomeration on the surfaces of the tested materials, with a higher NF percentage (10%) than a lower one (3%), indicating inadequate dispersion and distribution of the NPs within the matrix (Figs 1–5).

**Fig 6:**
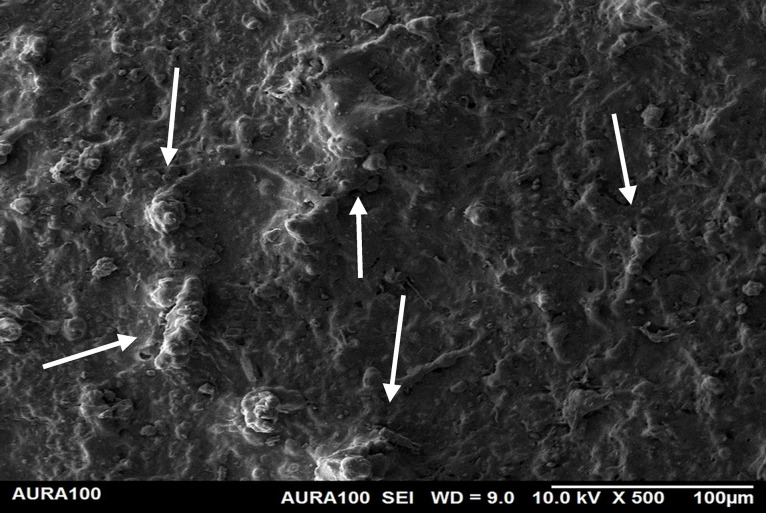
The surface roughness of the weathered MFSE with No NF.

**Fig 7:**
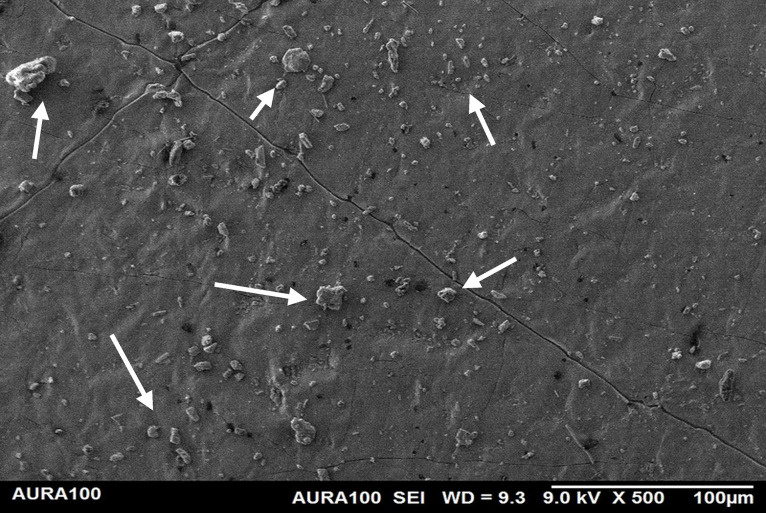
The surface roughness of the weathered MFSE with 3% SiO_2._

**Fig 8:**
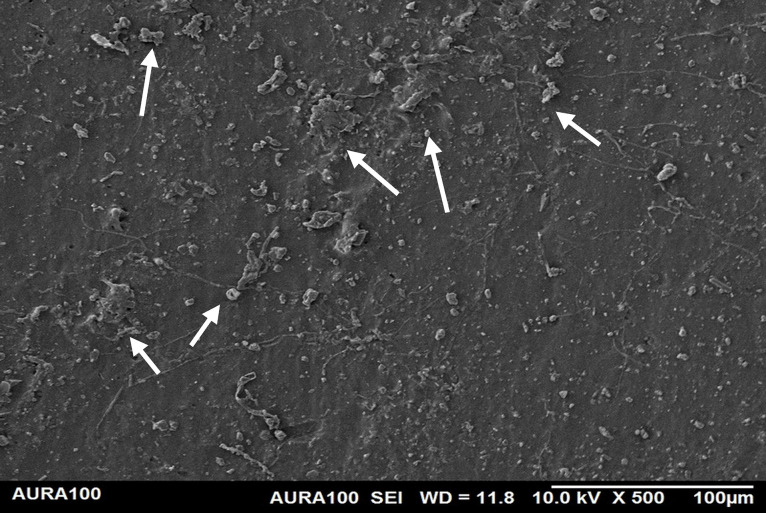
The surface roughness of the weathered MFSE with 10% SiO_2._

**Fig 9:**
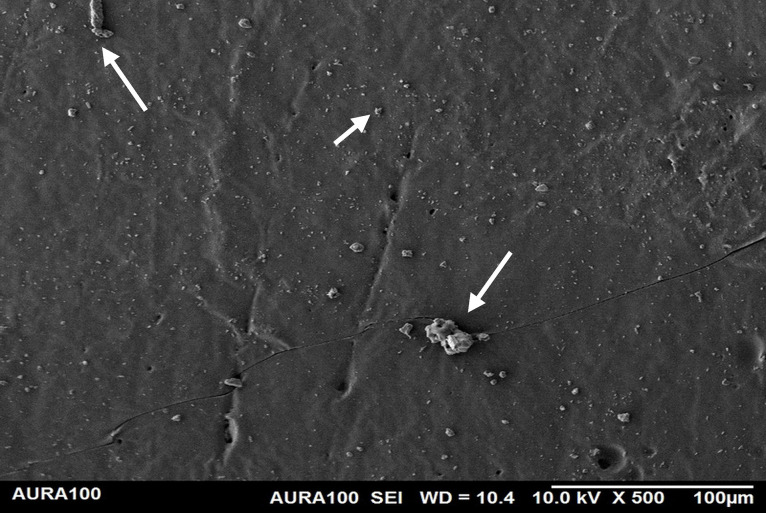
The surface roughness of the weathered MFSE with 3% TiO_2._

**Fig 10:**
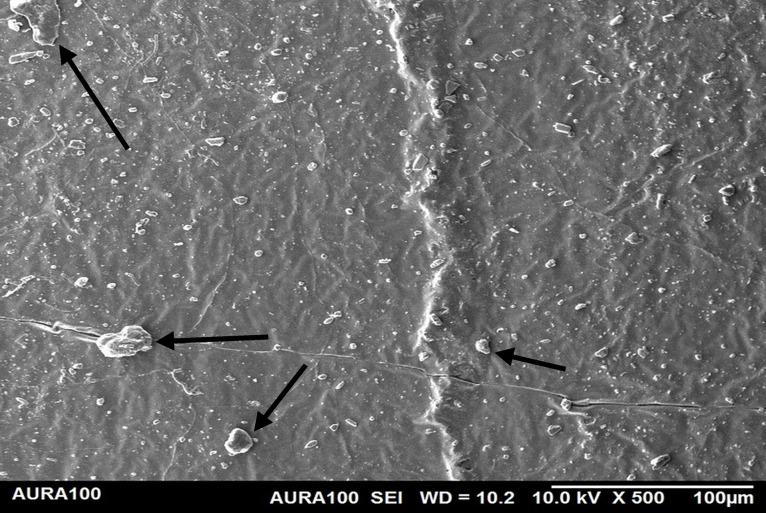
The surface roughness of the weathered MFSE with 10% TiO_2._

Regarding the weathered MFSE, the silicone containing 10% SiO2 and TiO2 had the lowest roughness, with no significant differences among the NFs (*P* = 0.37). There were no significant differences among MFS with 10% TiO_2_ and other specimens, including the MFSE with no NFs (*P* ≥ .37). Following two years of weathering, all test materials demonstrated a significant decrease in surface roughness (*P* < .001). Using SEM, the MFSE with different NFs exhibited lower surface roughness than the unfilled samples, which showed higher roughness. SEM also showed less agglomeration of NF on the matrix surface, indicating adequate dispersion and distribution of NF with the matrix (Figs 6–10).

### Surface hardness

Table 5 shows the test of normality (Shapiro-Wilk) for both surface hardness. The test indicates that the data distribution is normal, with no significant deviation from normality.

**Table 5:** Test of normality (Shapiro-Wilk test) for the surface hardness.

	Materials	Shapiro-Wilk		
variable		Statistic	df	Sig.
Surface_Hardness	NonWeathered_(nonfilled)	.913	8	.376
	Nonweathered_(3%)_Si_filled	.899	8	.281
	NonWeathered_(10%)_Si_filled	.929	8	.503
	NonWeathered_(3%)_Ti_filled	.924	8	.465
	NonWeathered_(10%)_Ti_filled	.945	8	.664
	Weatherd_Nonfilled	.909	8	.345
	Weathered_(3%)_Si_filled	.871	8	.155
	Weathered_(10%)_Si_filled	.930	8	.515
	Weathered_(3%)_Ti_filled	.939	8	.600
	Weathered_(10%)_Ti_filled	.939	8	.599

Table 6 shows the analysis of variance (ANOVA) confirms strong evidence of significant differences in surface roughness between groups (SS = 263.7, F = 100.2, P < .001, and η2 = .93). The effect is practically significant, with nearly 93% of the variability attributed to group differences. Table 7 shows the comparison of hardness, as shown in Table 3, for both weathered and non-weathered samples. Adding 10% SiO2 NF resulted in a significant increase in hardness, followed by 3% SiO_2_ (*P* < .001). Adding different percentages of TiO_2_ NF showed no significant differences in the hardness compared to non-filled silicone (*P* ≥ .2). The hardness of all tested specimens significantly increased after two years of weathering compared to those not subjected to the hot and humid weather (*P* < .001).

**Table 6:** Analysis of variance and mean squares in between and within groups in terms of surface hardness.

ANOVA					
Surface_Hardness					
	Sum of Squares	df	Mean Square	F	Sig.
Between Groups	2373.302	9	263.700	100.151	.000
Within Groups	184.311	70	2.633		
Total	2557.613	79			

**Table 7:** The hardness (Shore A) results of the study.

Filler	Non-weathered (Mean±SD) Shore A	Weathered (Mean±SD) Shore A	P-value Tukey's test
No NFs	23.2 ± .55^**[cd]^[z]^**^	30.7 ± 2.4^**[cd]^[y]^**^	<.001*
3% SiO2	28.0 ± 3.02^**[b]^[z]^**^	35.7 ± 1.1^**[b]^[y]^**^	<.001^*^
10% SiO2	32.7 ± .34^**[a]^[x]^**^	40.5 ± .56^**[a]^[y]^**^	<.001*
3% TiO2	22.3 ± 1.2^**[d]^[x]^**^	29.3 ± 1.6^**[c]^[y]^**^	<.001^*^
10% TiO2	24.6 ± .31^**[c]^[y]^**^	32.5 ± 2.4^**[d]^[y]^**^	<.001^*^
P-value	< 0.001^**$**^	< 0.001^**$**^	

^**[a,b,c,d]**^The comparison among different groups, while ^**[a]**^ is the highest, ^**[d]**^ is the lowest, and the same letters show no differences.

^**[y,z]**^There are significant differences within the same group, while ^**[x]**^ is the highest and ^**[z]**^ is the lowest. The same letter/s means no significant differences between the variables

*There are significant differences within the same group.

^**$**^There are significant differences among different groups.

### Analyzing the chemical reaction using FTIR

**Fig 11:**
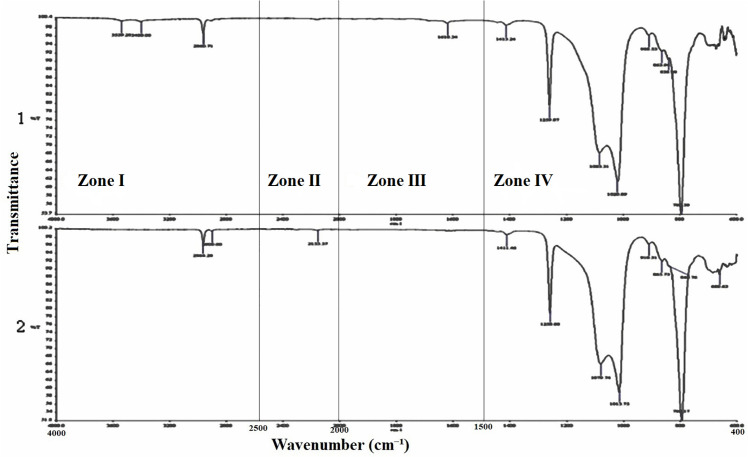
FTIR analysis of MFSE with No NF before (1) and after weather (2), showing the zone of wavenumbers. The main Peaks are concentrated in the fingerprint zone.

**Fig 12:**
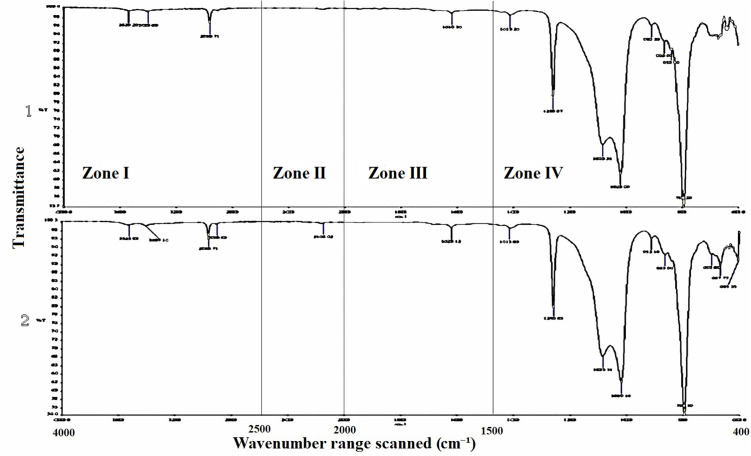
FTIR analysis of MFSE with 10% SiO_2_ NF before (1) and after weather (2), showing the zone of wavenumbers. The main Peaks are concentrated in the fingerprint zone.

**Fig 13:**
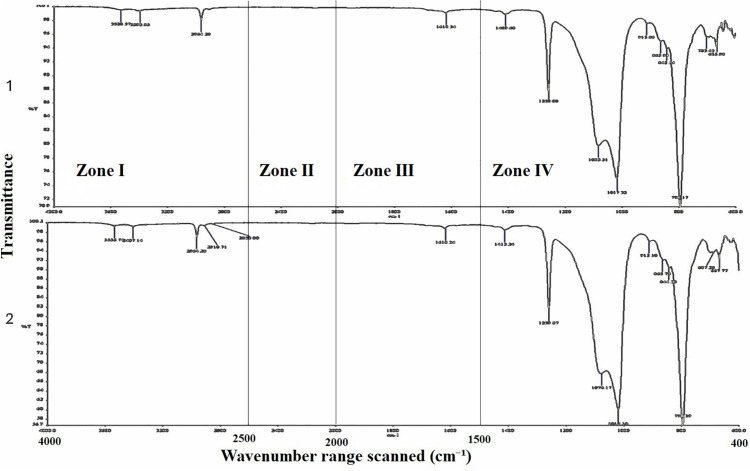
FTIR analysis of MFSE with 10% TiO_2_ NF before (1) and after weather (2), showing the zone of wavenumbers. The main Peaks are concentrated in the fingerprint zone.

When analyzing the chemical reactions that may occur between NFs and components of MFSE, the FTIR analysis showed that a similar reaction occurred between the different NFs and the silicone matrix (Figs 11–13). In the key single-bond region (wavenumbers between 2500 and 4000 cm-1), there were two narrow peaks (except for the MFSE with no NF, which has only one peak), indicating the absence of hydrogen bonding in the materials. In the triple bond region, with wavenumbers ranging from 2000 to 2500 cm-1, no peaks were observed, indicating no bonds or interactions in the materials. Similar to the triple bond region, the double bond region (1500−2000 cm-1) showed no peaks, indicating no bond or interaction. In the fingerprint region, which ranges from 400 to 1500 cm-1, three sharp peaks at 600, 800, and 1000 cm-1 were observed, indicating absorption bands for Ti-O, O-Ti-O, Si-O, and O-Si-O bonds.

## Discussion

The hot, humid weather and the incorporation of both SiO2 and TiO2 NF at low and high percentages significantly altered the surface roughness and hardness of the A-2000 MFSE after two years of outdoor natural weathering, leading to the rejection of the first null hypothesis. On the other hand, there was no chemical interaction between the tested nano-fillers and the silicon elastomers, which confirmed the second null hypothesis.

Maxillofacial silicone elastomers have proven efficient and effective in restoring maxillofacial defects, owing to their superior texture, durability, and color stability [1,5,7]. However, their short shelf life, rapid deterioration in physical and mechanical performance, color instability, and difficulty in repair are shortcomings [26]. An MFSE that has been in clinical use for over two years is required for patient satisfaction and function [40]. That is why the authors chose to conduct the study for two years. From another perspective, adding fillers at varying percentages has been shown to improve the mechanical properties of some MFSE [15,41–43]. Due to their high surface-to-volume ratio, tiny size, and active interaction within the polymer matrix, adding 2–3% nanosized SiO_2_ and TiO_2_ resulted in MFEs with superior physical, mechanical, and biological characteristics compared to their macroparticle counterparts. However, these NFs were applied to a limited number of MFSEs at ratios of 2–5% [15,41–43]. Additionally, adding a high NF content warrants further investigation, as it may affect the physical performance of these materials [19]. The authors chose to investigate the effects of weather and high NF concentrations on surface roughness and hardness, as these surface characteristics are crucial for maintaining the surface irregularity and natural softness of MFSE.

Malaysia experiences a tropical climate characterized by consistently hot temperatures (29–34°C), high humidity (80–90%), and year-round rainfall. The climate shows a distinct wet season during the November-January monsoon influenced by the east coast. That climate was devastated by extremely high UV radiation, with values >8 (10–14). UV radiation severely degrades medical silicones, leading to frequent replacement mainly due to discoloration and compromised physical properties [11,13,44]. The UV light generates free radicals that react with oxygen, producing oxy- and peroxy-radicals. These radicals break polymer chains (chain scission) and form cross-links. Consequently, molecular weight decreases as volatile degradation products form, degrading the material's physical and mechanical properties by increasing roughness and hardness of the prostheses, making insertion and removal, and hygiene difficult [45].

Surface roughness is a crucial characteristic of maintaining the surface irregularity of MFSE. The smoother the surface, the better the cleanability and the fewer micro-organisms retained, while the rougher the surface, the greater the retention and the more it mimics skin texture and wrinkles. Generally, the determination of a prosthesis's roughness lies in the hands of the prosthodontist, who relies on each patient's skin texture. The current study demonstrated that adding NFs decreased the surface roughness of A-2000 MFSE. With more NFs, smoother MFSE surfaces resulted. That was consistent with another study that found that adding different NF to other MFSE results in decreased surface roughness [46]. The SEM-captured figures also confirmed this result. Although the surface roughness before and after adding the different NFs was below the normal range (6–14 μm) [3,4], this allows the clinician and technician to introduce changes, such as pitting and roughening the surface, without increasing roughness beyond normal levels.

Regarding the weather, the hot, humid conditions reduced the surface roughness of all tested specimens, as reported in other studies [13,23]. The decrease in roughness is possibly due to time and UV exposure, which result in the redistribution and dispersion of agglomerated NF within the matrix. In the weathered specimens, both NFs significantly decreased surface roughness, with no significant difference between them, suggesting that their presence on the elastomer surface contributed to this effect. This agreement was supported by other studies demonstrating lower surface roughness of weathered MFSE than non-weathered [10,47].

The MFSE should exhibit adequate hardness within the range, as extremely hard prostheses may compromise performance and cause patient discomfort during insertion and removal, while softer prostheses may impact retention and performance. The Shore-A hardness value of MFSE was recommended to be 25–35 to mimic the texture of the thin skin covering structures very close to the bone, such as the orbit, nose, and ear areas of the maxilla [29,30]. However, these hardness values may be influenced by factors such as defect size and depth, proximity to the underlying bone, and patient factors. So, mimicking the hardness of bone or tissue alone may disturb the patient. As a result, the onus of selecting the appropriate hardness value rests solely on the clinician, in the patient's favor. Adding NF resulted in a significant increase in MFSE hardness. As the percentage of NF increased, the hardness increased. That was confirmed by other studies reporting different NFs with similar MFSE and vice versa [39,48]. The highest hardness was recorded by the high percentage of SiO_2_ (32.3), followed by the low percentage (27.8). That may favor the MFSE with 3% SiO_2_ in terms of surface roughness compared to those with TiO_2_ or no NF.

After outdoor weathering, the hardness of the weathered samples increased compared to that of the non-weathered samples. The samples with 10% SiO_2_ demonstrated the highest hardness (40, unfavorable, as mentioned before), while those with 3% SiO_2_ increased the hardness to 35.7, raising a question about its use, as it lies on the border of extreme hardness. Regarding the TiO_2_ NPs, although both concentrations showed surface hardness below the ideal level, after exposure to natural weather and UV, their surface hardness increased to within the recommended range. That agrees with a previous study that studied the influences of titanium on the surface hardness of another MFSE [29]. The increase in the surface hardness of SiO2 compared to TiO2 may be attributed to the surface characteristics of the SiO2 NFs, which exhibit a porous, spherical structure, whereas TiO2 shows anatase nanostructures [49,50]. That supports adding 10% TiO_2_ and 3% SiO_2_ NFs to the MFSE, indicating a higher surface hardness than materials with 10% SiO_2_ and 3% TiO_2_.

Analysis of the MFSE specimens using the FTIR spectrophotometer yielded spectra that were quite similar, with bands assigned to CH2 symmetric and asymmetric stretching vibrations in the single-bond zone (2500–4000 cm^−1^). Additionally, there were broad, strong three peaks at approximately 600, 1000, and 1200 cm − 1, ascribed to the absorption bands of Ti(Si)–O and O–Ti(Si)–O. The results of FTIR analysis confirmed the presence of hydroxyl groups on the surface of all elastomers. Therefore, based on current knowledge, a greater number of surface hydroxyl groups may enhance the hydrophilicity of elastomers.

One of the significant shortcomings of this study is that the in vitro setting excludes the patient's sweating, sebum, and saliva secretion, as well as the muscles’ action on the prostheses. Another limitation is the use of dental stone to prepare the mold for the specimens. This results in increased surface roughness of the materials when tested with the SEM. Using the conventional SEM alone to evaluate surface roughness and the regular FTIR method to evaluate the chemical reaction (due to availability) was not ideal for the study; it was preferred to add additional methods, such as Atomic Force Microscopy, FTIR-ATR, and a 3D profilometer. However, the method was unified with all test materials. Furthermore, the absence of color stability or tensile strength assessment may limit the generalizability of findings. Agglomeration at higher filler content, as observed via SEM, may be addressed in future studies via surface-modified nanofillers or dispersion aids. The potential long-term biological effects of NFs, such as cytotoxicity and biofilm adhesion, warrant further investigation.

## Conclusion

Despite the limitations of this in vitro study, the following can be concluded.

1. Adding 3% SiO_2_ and TiO_2_ NFs improved the surface properties of A-2000 MFSE by decreasing surface roughness and increasing hardness.
2. Adding 10% SiO_2_ NFs to the MFSE significantly increases hardness beyond the recommended limits, potentially affecting the insertion and removal of the maxillofacial prosthesis.
3. Adding 10% TiO2 NF did not result in additional statistically significant improvements and may lead to filler agglomeration.
4. Two years of natural, hot, humid weathering reduce surface roughness and increase the hardness of A-2000 MFSE.

## Supporting information

S1 File SR and SH (Raw data). (XLSX)
